# Recent findings on the role of wild-type and mutant p53 in cancer development and therapy

**DOI:** 10.3389/fmolb.2022.903075

**Published:** 2022-09-26

**Authors:** Mehregan Babamohamadi, Esmaeil Babaei, Burhan Ahmed Salih, Mahshid Babamohammadi, Hewa Jalal Azeez, Goran Othman

**Affiliations:** ^1^ Department of Biology, School of Natural Sciences, University of Tabriz, Tabriz, Iran; ^2^ Interfaculty Institute for Bioinformatics and Medical Informatics (IBMI), University of Tübingen, Tübingen, Germany; ^3^ Department of Medical Laboratory Technology, Erbil Health and Medical Technical College, Erbil Polytechnic University, Erbil, Iraq; ^4^ Department of Medical Laboratory Technology, AlQalam University College, Kirkuk, Iraq; ^5^ Student Research Committee, Faculty of Pharmacy, Tabriz University of Medical Sciences, Tabriz, Iran

**Keywords:** wild-type p53, mutant p53, MDM2, erastin, accumulation of p53, mTOR, therapeutic-approaches

## Abstract

The p53 protein is a tumor suppressor encoded by the TP53 gene and consists of 393 amino acids with four main functional domains. This protein responds to various cellular stresses to regulate the expression of target genes, thereby causing DNA repair, cell cycle arrest, apoptosis, metabolic changes, and aging. Mutations in the TP53 gene and the functions of the wild-type p53 protein (wtp53) have been linked to various human cancers. Eight TP53 gene mutations are located in codons, constituting 28% of all p53 mutations. The p53 can be used as a biomarker for tumor progression and an excellent target for designing cancer treatment strategies. In wild-type p53-carrying cancers, abnormal signaling of the p53 pathway usually occurs due to other unusual settings, such as high MDM2 expression. These differences between cancer cell p53 and normal cells have made p53 one of the most important targets for cancer treatment. In this review, we have dealt with various issues, such as the relative contribution of wild-type p53 loss of function, including transactivation-dependent and transactivation-independent activities in oncogenic processes and their role in cancer development. We also discuss the role of p53 in the process of ferroptosis and its targeting in cancer treatment. Finally, we focus on p53-related drug delivery systems and investigate the challenges and solutions.

## Introduction

The TP53 gene encodes a tumor suppressor protein called P53. The p53 protein was first identified in 1979 as a cellular protein that binds to the large T antigen of the simian virus (SV40) and accumulates in the nucleus of cancer cells. This protein contains domains of transcriptional activation, DNA binding, and oligomerization. The encoded P53 protein responds to various cellular stresses to regulate the expression of target genes, thereby causing DNA repair, cell cycle arrest, apoptosis, metabolic changes, and aging. Mutations in this gene and the functions of the wild-type p53 protein (wtp53) have been linked to various human cancers (Hofseth et al., 2004; Duffy et al., 2017).

**FIGURE 1:**
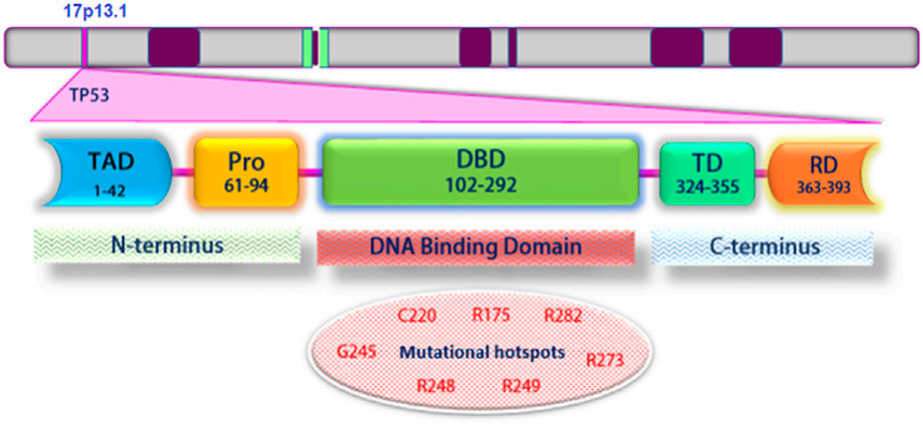
Schematic structure of TP53 and its different domains. Mutations frequently occur within the DNA-binding domain. Mutant codons are shown in red: Transcriptional activation domain (TAD); Proline-rich domain (PRD); DNA binding domain (DBD); Tetramerization domain (TD); Regulatory domain (RD).

According to studies, Eight TP53 gene mutations are located in codons that make up 28% of all p53 mutations. Mutations in these alleles appear in many human cancers and tissues. These alleles are called hotspots (Figure 1). Mutations in p53 gene hotspots are seen in human cancers for four reasons; one, proteins produced by mutant hotspots alleles have a highly altered structure. Two, mutagenic agents environmentally cause-specific allelic changes in the p53 gene. Three, these gene mutations are caused by specific DNA sequences. The performance loss and the changes in p53 mutant proteins are also seen. Hence, some of these mutant proteins have specific allelic functions that cause cancer (Baugh et al., 2018). The researchers found that the key to understanding the pathobiological activity of P53 was the false mutations found in the original TP53 cDNA clones. The ability of p53 to form tetramers allows the protein to behave predominantly negatively so that the p53 mutant allele suppresses wild-type p53 activity. Hence, some mutations in p53 may cause oncogenic activity (Scheffner et al., 1990; Sigal and Rotter, 2000).

The p53 protein is vital in maintaining DNA stability and preventing cancer. Normally, wild-type p53 protein (wtp53) binds to a negative regulator and is inactivated or degraded. However, when DNA damage occurs in a cell, the p53 protein is induced, causing the cell cycle to stop. This gives the cells a chance to repair, but if the damage is severe, the cells will develop apoptosis. The p53 (mtp53) gene mutation has been linked to various cancers Many mutations occur in the DNA binding domain of the p53 gene. On the other hand, the successful interaction of wtp53 protein with transcription factors facilitates the repression or activation of appropriate target genes. This process is disrupted by the presence of the defective mtp53 protein. Thus, converting mtp53 protein to wild-type p53 protein could be a promising way to prevent or reverse tumor progression (Zilfou and Lowe, 2009; Berke et al., 2022).

Among these, we can mention some small molecular compounds, such as RITA (RNA-induced transcriptional activation), which target p53. This molecular compound directly binds to p53, disrupts the interaction between p53 and MDM2, and prevents p53 degradation. RITA increases tumor suppressor expression and suppresses oncogene expression through p53 reactivation; As a result, it causes cell death mediated by p53 (Valente et al., 2018).

The p53 is a biomarker for tumor progression and a perfect target for designing cancer treatment strategies. In wild-type p53-carrying cancers, abnormal signaling of the p53 pathway usually occurs due to other unusual settings, such as high MDM2 expression. These differences between cancer cell p53 and normal cells have made p53 one of the most important targets for cancer treatment (Zhang et al., 2022).

In recent years, studies in this field have shown the structure, function, and role of p53 in tumor formation and development. These basilar studies strongly support the design and development of new approaches to targeting the mutant and wild-type p53 in cancer treatment. For example, delivery of wild-type p53 by adenovirus infection kills cancer cells and affects and suppresses tumor growth in preclinical and clinical trials (Valente et al., 2018). Also, methods such as chemotherapy, radiotherapy, and immunotherapy with specific targeting of the mutant p53 to suppress tumor growth are becoming a promising approach in the treatment of cancer (Beckta et al., 2014; Huang, 2021). Some plant substances and compounds are effective on wild-type and mutant p53 by inducing apoptosis or preventing cell proliferation, for example, Gemini curcumin and dendrosomal nano-curcumin in colorectal and breast cancer cell lines (Sobhkhizi et al., 2020; Ebrahimi et al., 2021).

## Structure of wild-type p53 and mutations leading to cancer

The p53 protein consists of 393 amino acids, with four main functional domains: transcription, DNA binding, tetramerization, and regulatory (Figure 1). Also, this protein includes five protected regions under the headings (I, II, III, IV, and V) and the loop-helix structure (L, S, and H). Highly protected domains overlap with loop domains and are part of the protein’s three-dimensional structure. In addition, there is a strong association between mutations and p53 three-dimensional structural domains (May and May 1999). Typically, wild-type p53 loses its function with a single-point mutation. This mutation causes a change in the structure of the core DNA binding domain of the protein (conformational mutation) or a change in the DNA binding capacity (contact mutation). Recent findings suggest that p53 mutants and their fragments can form protein masses both *in vitro* and *in vivo* (Higashimoto et al., 2006; Xu et al., 2011). Accumulation of p53 in both contact and conformational mutations in samples taken from patients’ tumor tissue has been observed in several cancer cell lines; this indicates an association between mutated p53 accumulation and tumor growth (Kanapathipillai, 2018).

In most human cancers, p53 hot spot mutations (both conformational and structural) are observed at the amino acid sites 175, 245, 248, 249, 273, and 282 (Figure 1) (Freed-Pastor and Prives, 2012). Meanwhile, R248Q, R248W, and R175H mutations showed p53 protein accumulation in different tumor samples, while p53 protein accumulation was not reported in tumor samples containing R273H and R249S hot spot mutations (Soragni et al., 2016; De Smet et al., 2017).

As mentioned, two hotspots and common cancer mutations in p53 protein are amino acid sites 175 and 273. R175H and R273H are more prone to aggregation than wild-type (WT) p53, and their pathological aggregation can lead to various cancers. A recent study investigated the dynamic and structural properties of the R175H and R273H mutants in the p53 core domain (p53C) using extensive all-atom molecular dynamics simulations. In this regard, the researchers found that in both R175H and R273H mutations, the β-sheet structure is well preserved; however, a larger hydrophobic surface and a higher loop flexibility than WT p53C were seen, thus predisposing proteins to accumulate in the cell. Also, an allosteric pathway has been identified through which the R273H mutation increases the flexibility of the N-terminal region around loop 2. These results indicate mechanical insights into the high accumulation tendencies of R175H and R273H mutants (Bykov et al., 2018; Li et al., 2020).

## The loss of wild-type p53 function in oncogenic processes and cancer development

In cancer biology, the vital point about p53 is that; the p53 mutant protein is found in 50% or more of 50% of human cancers. In addition to losing function, the mutant p53 can have a dominant-negative effect on the remaining wild-type p53 allele and subsequently inactivate it by losing heterozygosity (LOH). Also, some p53 mutations have additive functions, which will cause the tumor to grow. In cancers in which wild-type p53 is conserved, it is usually in regulatory genes that encode the up or down pathways of p53; changes are observed (Steels et al., 2018).

Among p53 mutants, missense mutations not only cause the mutated p53 protein to lose its wild-type (LOF) function and gain dominant-negative activity but also increase the function of the mutated p53, leading to the tumor’s more aggressive behavior and drug resistance (Zhang et al., 2022).

In the remainder of this section, we will discuss the relative contribution of wild-type p53 loss of function, including transactivation-dependent and transactivation-independent activities in oncogenic processes and their role in cancer development.

### The role of p53 protein in tumor suppression, transcription factor, and stress sensor; transactivation-dependent activities

The TP53 gene acts as a tumor suppressor and cellular stress sensor. In stress-free cells, p53 is targeted for degradation by E3 ubiquitin ligase MDM2 and kept at low levels. Stress signals, such as DNA damage, oncogene expression, and hypoxia relieve P53 from MDM2 inhibition. Mutations in human cancers that occur mainly in the domain of DNA binding and specific sequences; disrupt p53 activation. A mutation in the TP53 gene disrupts the binding capacity of DNA by disrupting the structure of the p53 protein; it also increases oncogenic function. The TP53 gene also encodes a transcription factor that is an essential factor in cancer prevention. Inactivation of this gene leads to the most common mutation in sporadic human cancers (Boutelle and Attardi, 2021).

Studies have shown that p53-R248Q expression reduces the motility and invasiveness of lung and breast cancer cells in a p53 transactivation-dependent manner. Indeed, in the cell lines expressing p53-R248Q, myosin light chain two expression decreases. This chain is a protein involved in actomyosin-based motility. Also, the expression of R248Q in Matrigel’s absence significantly reduces the migration rate in a trans-dependent manner (Olszewski et al., 2019).

**FIGURE 2:**
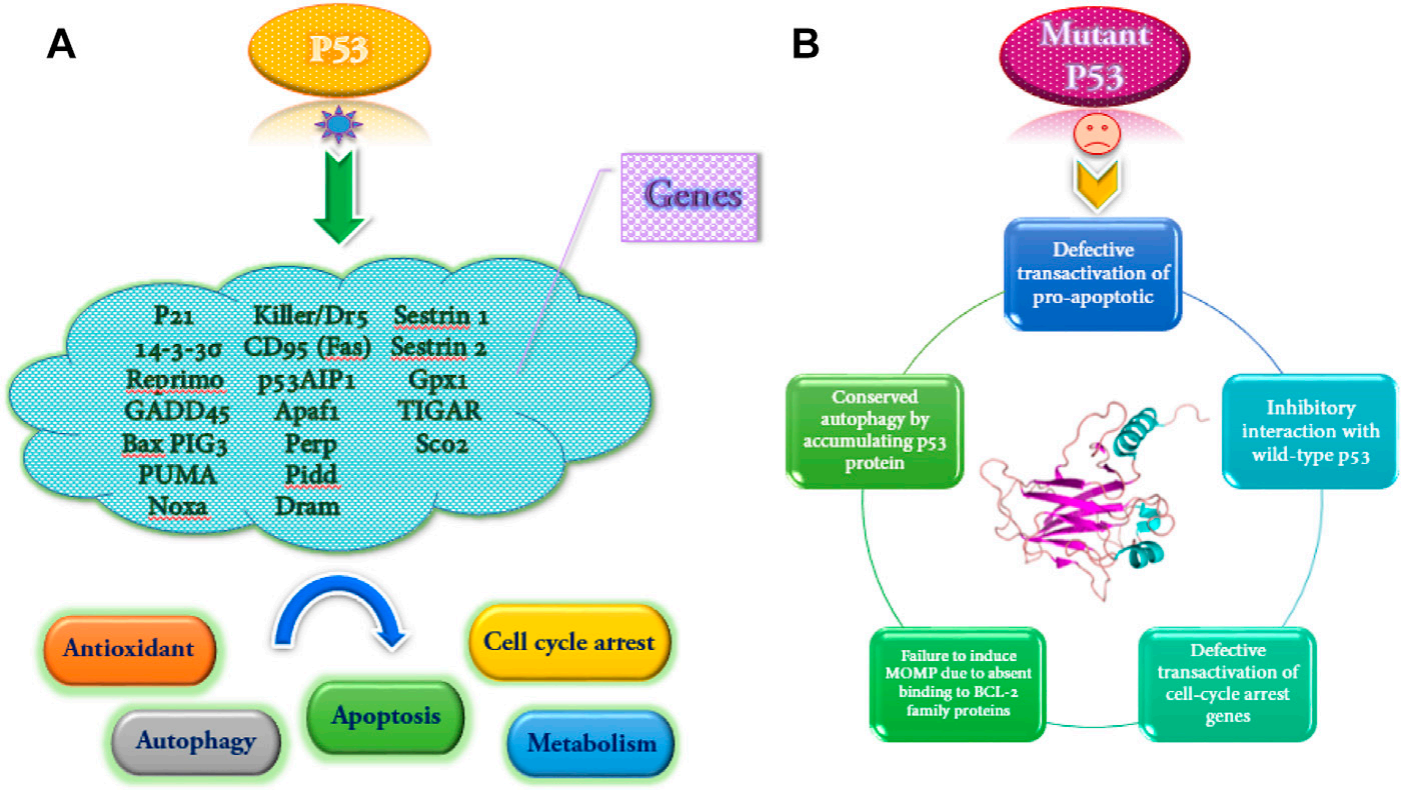
Some genes are transactivated by wild-type p53, and several functional consequences of p53 activation **(A)**. Functional implications of mutant p53 **(B)**.

The wild-type p53 regulates the expression of more than 2,500 genes as a transcription factor. The p53 helps respond to various stresses such as DNA damage, hypoxia, and oncogene activation. Also, depending on the severity and duration of stress, it leads to cell cycle arrest or apoptosis (Dehghan et al., 2015). Some of the genes that are activated by wild-type p53 and some functional consequences of wild-type p53 activation and the mutant type are shown in Figure 2.

The wild-type p53 dysfunction can disrupt autophagy signaling. In this regard, the nuclear transactivation-dependent activation of autophagy by p53 is often impaired due to inactivation or deficiency of p53 in the cytoplasm. However, the tumor can stimulate pro-autophagic functions. The downregulation of the mTOR set translates this activity. The researchers found that p53 may inhibit autophagy using direct protein-protein interactions. Based on the findings of a study, gain-of-function (GOF) mutant p53 proteins inhibit the autophagic pathway and increase the proliferation of pancreatic and breast cancer cells. This reaction has been accompanied by stimulation of AMPK-mTOR genes and suppression of DRAM, Beclin-1, ATG12, and sestrin genes (Cordani et al., 2016; Mrakovcic and Fröhlich, 2018).

### Another dimension of tumor suppression: p53 functions independent of transactivation

Although most studies on p53-mediated tumor suppression mechanisms are based on the activation of p53 target genes, other aspects of this issue include the performance of p53 independent of the reaction, which has been reported recently. Induction of apoptosis in mitochondria can be considered the best function of p53 independent of transactivation. The p53 protein can bind members of the BCL-2 family of anti-apoptosis to replace them with members of the BCL-2 family of pro-apoptotic. It can also directly activate BAK and BAX, infiltrate the external mitochondrial matrix, and stimulate apoptosis (Table 1) (Ho et al., 2020).

**TABLE 1:** The function of transactivation-independent p53.

Cellular position	Effects	References
Mitochondria	Induction of apoptosis	Ho et al. (2020)
	Direct activation of BAX and BAK	
	Binding of anti-apoptotic BCL-2 family members	
	Infiltration into the external matrix	
Cytoplasm	Centrosome duplication	Green and Kroemer, (2009), Jiang et al. (2011)
	Apoptosis induction *via* MOMP	
	Restriction of tumor metabolic pathways	
	Inhibition of autophagy	
Nuclear	Transrepression	Green and Kroemer (2009), Ho et al. (2020)
	Homologous recombination	
	DNA replication	
	Increase genomic integration	

The p53 protein, in addition to mitochondria, has functions other than transactivation in other sites, such as the cytoplasm and nucleus (Table 1), which can help suppress the tumor. In the cytoplasm, the p53 protein can bind and inhibit the rate-limiting enzyme of the pentose phosphate pathway, G6PD, thereby restricting the metabolic pathway leading to the tumor (Jiang et al., 2011). The p53 protein in the nucleus can also increase genomic integrity to prevent excessive and erroneous recombination. P53 does this by various methods, such as limiting the motility of transposons and other classes of repetitive elements and binding the homologous recombination protein RAD51. These findings suggest that activation-independent performance of P53 may be necessary for some settings in tumor suppression, and future trials will shed light on the importance of this in tumor suppression (Ho et al., 2020).

In a study of mouse models of intestinal cancer based on the Wnt signaling pathway caused by an ApcMin/+ mutation or deletion of Csnk1a1 in the proximal intestine, cancer was suppressed in the presence of p53R172H. It can be said that this repression is based on a mutation in the DNA binding domain that is responsible for the trans-activation of the target gene and is related to the p53 −/− model. This is related to the inhibition of TCF4 chromatin binding in the Wnt pathway and, in a sense, the suppression of this pathway and tumor-independent p53 activation. Further tests are needed to prove this mutant’s neomorphic activity or presence in wild-type p53 (Kadosh et al., 2020).

Based on the analysis of knock-in mouse models in another study, which expresses a mutation in p53R178E, The mutation P53R178E has been shown to behave like an empty p53 allele in both spontaneous and Eμ-Myc mouse tumorigenesis. This mutant retains apoptotic activity in chemotherapy in Eμ-Myc mouse lymphoma and is MDM2-deficient in growing embryos *in vivo*. p53R178E may provide a potential mechanism for apoptosis independent of the transactivation induced by p53R178E, and it can localize in the mitochondria (Timofeev et al., 2019).

The MDM2 is commonly known as the primary negative regulator of p53, but evidence suggests that MDM2 has functions independent of its role in regulating p53. Disruption of the regulation of these p53-independent functions could underlie the oncogenic property of MDM2, which is seen in the absence of p53. These findings explain why about 10% of human tumors overexpress MDM2 instead of inactivating p53 through other mechanisms. Since the p53-independent functions represent new targets for potential therapeutic interventions, the finding of these cellular roles, including the function of MDM2 in pathogenesis, will be important in tumor biology studies and cancer treatment (Bohlman and Manfredi, 2014). MDM2 is generally thought to suppress P53 by two distinct mechanisms, one is to cover p53 access to the transcription machinery by connecting to the N-terminal domain of p53, and the other is to target it for proteasomal degradation and ubiquitinating p53 (Pei et al., 2012).

## The role of tumor suppressor protein p53 in the regulation of ferroptosis

When p53 stops the G1 cell cycle and facilitates DNA repair or apoptosis to kill damaged cells, it can be considered the best description of p53 function in response to acute DNA damage (Boutelle and Attardi, 2021). In addition to known activities of wild-type p53, including effects on checkpoints, cell cycle arrest, and apoptosis, there is recent evidence of the importance and role of other mechanisms in tumor suppression. Ferroptosis has been highly regarded as an iron-dependent method that causes non-apoptotic cell death defined in 2012. Unlike apoptosis, p53 activation alone is insufficient in cell death by direct induction of ferroptosis. It differs from apoptosis, necrosis, and other types of cell death in genetics, morphology, metabolism, and molecular biology. (Zhao et al., 2020; Liu and Gu, 2022).

**FIGURE 3:**
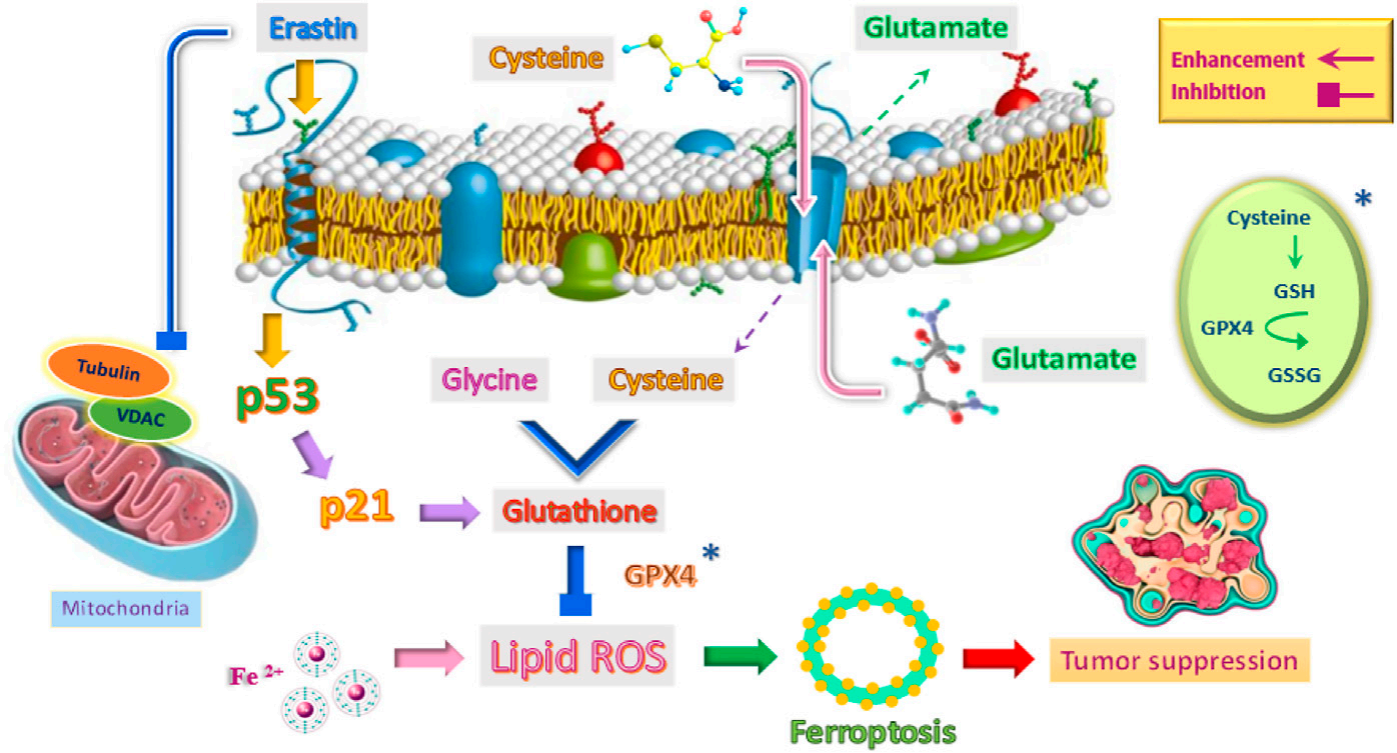
Induction of ferroptosis mechanism to suppress tumor by emphasizing the role of p53 and other factors.

Ferroptosis is a type of cell death that occurs following lipid peroxidation and reactive oxygen species (ROS) that can be regulated in various ways, including levels of transcription factors and changes in the activity of antioxidant enzymes. The wild-type p53 regulates ferroptosis through lipid peroxidation, amino acid metabolism, and the biosynthesis of glutathione and phospholipids (Zhao et al., 2020; Ji et al., 2022). Also, through its metabolic targets, the p53 protein in the ferroptosis process can modulate this response in the presence of ferroptosis stimuli, such as high levels of ROS and GPX4 inhibitors (Figure 3). GPX4 is a member of the GPX family and is essential in maintaining cellular homeostasis. The critical point is that the pathways of wild-type P53-mediated ferroptosis can be effective in treating human cancers (Zhao et al., 2020; Liu and Gu, 2022).

It is important to note that p53 sometimes reduces cellular susceptibility to ferroptosis. Studies have shown that wild-type p53 protein activates p21 protein in a transcription-dependent manner and delays the onset of ferroptosis (Tarangelo and Dixon, 2018; Tarangelo et al., 2018). Researchers have also found that p53 in colorectal cancer cells (CRC) can play a role in inhibiting ferroptosis with the help of a compound called dipeptidyl peptidase 4 (DPP4) (Xie et al., 2017). To put it better, p53 plays a double-edged sword role in the ferroptosis process. On the one hand, it can increase cell susceptibility to ferroptosis, kill abnormal cells and prevent tumorigenesis. On the other hand, it can act as an inhibitor of the ferroptosis pathway. The role of p53 in the ferroptosis signaling pathway is complex. So far, this mechanism has not been thoroughly studied, and the specific function of p53 in this pathway and cancer treatment needs further study (Zhao et al., 2020). It is important to note that iron-based nanomaterials (such as iron-doped nanomaterials and iron oxide nanoparticles) can be used as anticancer agents. They stimulate ferroptosis in cells by over-producing ROS (Zhang et al., 2021).

### The effect of erastin on p53 and its outlook in cancer treatment

There are two main pathways in the induction of ferroptosis. The cysteine-glutamate transporter is the first pathway, which includes erastin, sulfasalazine, and glutamate. The second pathway in the induction of ferroptosis directly inhibits glutathione peroxidase (GPX) activity and includes RSL3 and DP17 (Yang et al., 2014; Liang et al., 2019). In the meantime, erastin is different from other stimulants of ferroptosis because it can stimulate several molecules and have an effective, rapid, and lasting effect (Figure 3). In other words, erastin does not determine a single path (Sato et al., 2018).

One of the roles of erastin is to influence the voltage-dependent anion channel (VDAC). This ion channel is located in the mitochondria’s outer membrane (Figure 3). VDAC controls molecular and ion exchange between mitochondria and cytoplasm (Mazure, 2017; Becker and Wagner, 2018). VDAC permeability can be altered with the help of drugs. This causes mitochondrial metabolic disorders, ROS production, and ultimately oxidative death (Maldonado, 2017).

Erastin as a tubulin antagonist, can alter the permeability of the outer mitochondrial membrane by opening the VDAC channel. In other words, erastin prevents VDAC from being blocked by cytoplasmic-free tubulin (Yagoda et al., 2007; DeHart et al., 2018). VDAC opening has three biological effects: increased mitochondrial metabolism, decreased glycolysis, and increased ROS production. Since inhibition of mitochondrial metabolism and glycolysis are metabolic features of cancer cells, increased VDAC opening by certain drugs and the production of ROS affects most cancer cells and can kill these cells or reduce their proliferation (DeHart et al., 2018; Fang and Maldonado, 2018). Because non-proliferating cells do not have the high levels of free tubulin that characterizes cancer cells, one of the therapeutic benefits of erastin as a VDAC-tubulin antagonist is that it specifically kills cancer cells. Therefore, erastin can be used as a new anticancer drug target by regulating metabolism (Reina and De Pinto, 2017). The critical point is that erastin can increase ferroptosis by activating P53. Wild-type p53 induces ferroptosis by inhibiting the activity of the X_C_^−^ system (Gnanapradeepan et al., 2018).

Studies have shown that after treatment of A549 lung cancer cells with erastin, p53 transcription products were significantly regulated, and ROS levels increased. According to the findings, p53 activation depends on ROS due to erastin exposure and activates the downstream p53 pathway (Huang et al., 2018).

In 2015, Jiang and colleagues at Columbia University Cancer Genetics Institute created p53^3KR^ mutant cells that lacked acetylation. These cells lost the function of wild-type p53 to induce apoptosis but still could inhibit *SLC7A11* transcription. *SLC7A11* is highly expressed in human tumors and inhibited by ROS-induced ferroptosis. The p53 protein inhibits cysteine uptake by inhibiting the expression of *SLC7A11*, a key anti-Porter component of cysteine/glutamate, and induces ferroptosis in cells. When erastin was used separately to treat p53-deleted cells, and p53^3KR^ mutant cells, p53-deficient cell mortality was reported to be very low (≤10%); This was while the p53^3KR^ mutant cell mortality was very high (>90%). However, if the expression of *SLC7A11* is excessive in p53^3KR^ mutant cells, treatment with erastin will significantly reduce the cell death rate (20%) (Jiang et al., 2015).

In another type of mutant p53, wild-type p53 function and the ability to inhibit *SLC7A11* transcription were lost. This mutant model, called p53^4KR98^, significantly reduces cell death and tumor inhibitory function during treatment with erastin. These results suggest that activating p53 by erastin may play an essential role in tumor inhibition by inhibiting transcription of *SLC7A11* and stimulating the ferroptosis process (Wang et al., 2016a).

It should be noted that the role of erastin in activating p53 and inducing ferroptosis is not applicable in all cells. However, it helps identify specific targets for inducing cancer cell death and inhibiting ferroptosis in normal cells to reduce the side effects of chemotherapy and provides ample scope for future research (Zhao et al., 2020).

### The role of radiotherapy in tumor suppression with effect on p53 in the mechanism of ferroptosis

Radiotherapy (RT) breaks down two strands of DNA and produces reactive oxygen species (ROS); this causes the cessation of the cell cycle, aging, and cell death (Azzam et al., 2012). Since the p53 is the most common mutated gene in human cancers, it is considered one of the main goals of radiotherapy. Hence, ferroptosis is a critical mechanism for mediating p53 function in tumor radiosensitivity. RT-mediated p53 activation reduces *SLC7A11* expression and induces RT-induced ferroptosis by suppressing glutathione synthesis. In other words, p53 deficiency increases the resistance of cancer cells or tumors to radiotherapy by inhibiting *SLC7A11*-mediated ferroptosis. Studies have shown that ferroptosis inducers (FINs) that inhibit *SLC7A11* display significant radiation sensitizing effects on tumor organoids and xenografts derived from patients with p53 deficiency or mutation. Accordingly, activation of p53 and RT-induced ferroptosis leads to better clinical outcomes for RT in cancer patients, and it is suggested that these FINs be used in combination with radiotherapy to treat p53 mutant cancers (Lei et al., 2021; Yang et al., 2022).

## Recent advances in the use of drugs to treat cancer by targeting p53 signaling pathways

The frequency of p53 mutations in most cancers is about 50%, but this frequency varies depending on the type of cancer. For example, p53 mutation has been reported in cancers of laryngeal (40.4%), esophageal (43.1%), head and neck (40.6%), ovarian (47.8%), and colorectal (43.2%). However, the frequency of p53 mutation in a small number of cancers is less than 20%. For example, endocrine (14.6%), hematopoietic (12.7%), and cervical (5.8%) tumors have the lowest frequency of p53 mutations (Olivier et al., 2010). Therefore, it is imperative that p53 mutations be targeted for drugs and that studies be developed in this field (Issaeva, 2019). Recent studies point to using designer peptides and poly arginine analogs to inhibit p53 accumulation and tumor growth by treating cancer as a protein accumulation disease (p53 protein accumulation). Also, the use of small stress molecules as potential anti-p53 accumulation drugs has been suggested (Table 2) (Kanapathipillai, 2018).

**TABLE 2:** A summary of therapeutic approaches to counteract the accumulation of mutated p53.

Anti-accumulation of p53	Type of effect	Type of tissue	*In-vitro*/*in vivo*	References
Designer peptide	Inhibit p53 mutant accumulation	Ovarian cancer	*in vitro* and *in vivo*	Soragni et al. (2016)
Arginine and analogues (Small stress molecules)	Inhibit p53 mutant (R248Q) mimetic peptide accumulation	lung cancer	*in vitro*	Chen et al. (2017)
	Inhibit p53 mutant (H719, R248Q) and cancer cell proliferation	lung cancer	*in vitro*	Chen et al. (2017)
	Inhibit p53 mutant (SK-BR-3, R175H) and cancer cell proliferation	Breast cancer	*in vitro*	Chen et al. (2017)
Acetylcholine chloride (Small stress molecules)	Inhibit p53 mutant (R248W) mimetic peptide accumulation	—	*in vitro*	Chen and Kanapathipillai, (2017)

Protein accumulation, instability, improper folding, and defective transport generally lead to cellular functions and processes dysfunction. Many of these protein accumulation diseases are caused by the wrong folding of the protein, possibly due to genetic mutations and environmental stress conditions (Brodsky, 2014; Valastyan and Lindquist, 2014). Accordingly, therapies for reactivation of wild-type p53, recovery of p53 downstream pathway function, and degradation of mutant p53 have been considered. It is important to note that these therapeutic strategies for inhibiting p53 accumulation are relatively new and require further research (Bossi and Sacchi, 2007; Wang and Sun, 2010).

### The role of designer peptides in preventing p53 protein accumulation

Based on recent studies, a peptide-based approach to inhibit p53 mutant accumulation has been considered. In this regard, the designer peptide, ReACp53, inhibits p53 mutant accumulation and suppresses tumors *in vitro* and *in vivo*. These peptides are for the region prone to p53 accumulation; amino acids 252–258 are designed. For example, research has been conducted on treating different models of ovarian cancer *in vivo* and *in vitro* and the potential of these peptides in accumulating p53 mutants (Table 2). This study showed that peptides could improve p53 function, inhibit p53 accumulation and stop tumor growth *in vivo* (Soragni et al., 2016; Bykov et al., 2018).

### The role of small stress molecules in preventing p53 protein accumulation

As mentioned, small stress molecules stabilize proteins under challenging conditions, and following the accumulation of peptides, they show inhibitory potential in neurodegeneration (Kanapathipillai et al., 2008). Small stress molecules such as mannosylglycerate (MG), ectoine and hydroxyectoine help to stabilize proteins in stressful situations and effectively inhibit peptide-related neurodegenerative diseases (Kanapathipillai et al., 2005; Ryu et al., 2008). On the other hand, cancers in which p53 mutates may have a common aggregation mechanism with neurological diseases. Also, based on the prion aggregate-like behavior of p53 mutant, there may be similarities between protein accumulation in neurological diseases and cancer. Based on this, it can be said that small stress molecules have the potential to modulate the accumulation of p53 mutants (Silva et al., 2014). According to studies, arginine can also be a small stress molecule in the breakdown and stabilization of proteins (Baynes et al., 2005).

Arginine analogues, including canavanine, citrulline, and ornithine, can stabilize proteins and be used as candidates for treating cancers caused by p53 accumulation. For example, mutant types R248Q and H719 in lung cancer and mutations SK-BR-3 and R175H in breast cancer prevent the proliferation of cancer cells (Table 2) (Chen et al., 2017). In this type of treatment, molecules can be delivered through appropriate drug carriers or nanotubes, which increases the half-life, effective targeting, and effectiveness of treatment. These studies need further investigation (Kanapathipillai, 2018). In addition to the above, other small stress molecules, including the cation molecules acetylcholine chloride, can inhibit the accumulation of the R248W mutant peptide and have anti-cancer properties (Table 2) (Chen and Kanapathipillai, 2017).

### The role of MDM2/MDM4 pharmaceutical inhibitors on the p53 mutant

One of the methods associated with P53 in cancer treatment is using pharmacological inhibitors of the p53-MDM2 interaction with dose-limiting thrombocytopenia. In addition, newer compounds such as dual MDM2/MDM4 inhibitors are under clinical trial (Zhao et al., 2015; Issaeva, 2019). Mutation and down reduction of wild-type p53 mediated by MDM2/MDM4 are the main mechanisms of p53 disruption (MDM2 and MDM4 are known as HDM2 and MDMX, respectively). Therefore, various p53-MDM2/MDM4 antagonists are undergoing clinical trials, the most important of which is idasanutlin, which is currently in the third phase of clinical trials in patients with recurrent or refractory acute myeloid leukemia. There are also reports of the impact of the two combinations APR-246 and COTI-2, which are mutant p53 activators. These compounds are MDM2/MDM4 inhibitors, and advances have accompanied research in this field, but in order to be clinically effective, more studies are needed (Issaeva, 2019; Duffy et al., 2020).

### The role of HSPs pharmaceutical inhibitors on the p53 mutant

Another essential item is the HSP90. This heat shock protein is involved in the accumulation of mutant p53 by inactivating the p53 ubiquitin ligands, CHIP and MDM2. Targeting the p53-HSP mutant complex, which results in the degradation of the p53 mutant protein, is one treatment strategy (Alexandrova et al., 2015). Studies have shown that non-small cell lung cancer (NSCLC) cells become sensitive to radiation by the HSP90 inhibitor ganetespib, and this has been associated with advances in clinical trials (Phase III Study) in NSCLC (Wang et al., 2016b; Pillai et al., 2020).

### The role of histone deacetylase inhibitors on the p53 mutant

The kevetrin (thiobutyronitrile), which has an inhibitory effect on histone deacetylase (HDAC), can directly affect mutant p53 and lead to p53 phosphorylation in serine 15 and cessation of cell division in the G2/M stage and finally tumor cell death. It should be noted that its role in inhibiting ovarian cancer cell lines is well known (Kumar et al., 2011; Kumar et al., 2017).

### Effect of zinc deficiency on wild-type p53 and its therapeutic solution

According to recent studies, zinc deficiency induces oxidative DNA damage and is involved in developing the mutated p53 (Hainaut and Mann, 2001; Ho and Ames, 2002). Researchers have investigated using thiosemicarbazones as zinc metallochaperones to reactivate mutated p53. To solve this problem, they proposed a two-step mechanism that activates the wild-type protein structure of p53 through post-translational modifications. Therefore, they first regenerated the zinc linker and then modified the mutant p53 that had lost its function (Puca et al., 2011; Yu et al., 2017; Kogan and Carpizo, 2018).

### The latest therapeutic achievements: Novel nanomedicines to counteract p53 mutations

Some of these methods have limitations and may not be used to treat all cancers associated with p53 accumulation. For example, in the designer peptide method, if wild-type p53 is somewhat compacted or unfolded, wild-type p53 structures may also be targeted. If this occurs in normal cells in the body, it can lead to systemic toxicity. Also, in therapies based on small molecules, peptides are rapidly eliminated from the bloodstream due to their short half-lives, small molecular weight, and size; as a result, more optimization is needed (Kanapathipillai, 2018). Hence, it would be ideal if nanotechnology approaches could be used to formulate small stress molecules in a latent drug carrier. One of the central delivery systems in cancer drug delivery is the use of nanoformulations. Nanoparticles have the potential to deliver the drug efficiently to the tumor site, do not affect normal tissues, have better plasma solubility, and produce successful therapeutic results. Also, this method increases the half-life of drug-carrying molecules, and the instability of these peptides is eliminated (Alexis et al., 2010; Kanapathipillai et al., 2014).

Another treatment method is the production of gold nanoparticles (AuNPs). These nanostructures provide a robust and non-toxic delivery system for gapmers in cancer cells and significantly reduce mutant p53 proteins. They overcome chemoresistance to gemcitabine. The formulation of AuNPs consists of a combination of a mixed polymer layer of polyethylene glycol (PEG) and PEI and a layer-by-layer assembly of bPEI through a sensitive linker. These nanoparticles can bind oligonucleotides through electrostatic interactions and release them as glutathione (García-Garrido et al., 2021).

The redox response for efficient delivery of p53-encoding synthetic messenger RNA (mRNA) is another nanoparticle (NP)-based engineered platform. Based on the findings, synthetic p53-mRNA nanoparticles significantly delay the growth of p53-null HCC and NSCLC cells by inducing apoptosis and cell cycle arrest. Also, p53 restoration significantly improves the sensitivity of these tumor cells to everolimus, a mammalian target of rapamycin (mTOR) inhibitor that is not expressed in HCC and advanced NSCLC. Furthermore, co-targeting the mTOR and p53 signaling pathways lead to distinct antitumor effects in several animal models of HCC and NSCLC *in vitro*. These findings suggest that combining synthetic mRNA NP delivery strategy with other cancer treatments can be very effective (Kong et al., 2019).

In addition to the above, monoclonal antibodies can be potent tools against p53-specific mutations and maximize treatment potential with personalized or precision medicine approaches (Hwang et al., 2018).

## Conclusion and outlook

Since the wild-type P53 protein has important and extensive roles and functions within the cell; as a result of mutations in this protein, which can occur due to various factors, causes various types of cancer. According to the findings, the p53 mutant protein is found in 50% or more of 50% of human cancers. Therefore, specialized therapeutic approaches to target P53 mutants or pathways associated with this protein have received much attention recently Although research in this area needs further investigation, it is hoped that it will lead to proper and effective treatments for various types of cancer and pave the way for future studies.
